# The Promotion of Physical Activity from Digital Services: Influence of E-Lifestyles on Intention to Use Fitness Apps

**DOI:** 10.3390/ijerph17186839

**Published:** 2020-09-18

**Authors:** Jerónimo García-Fernández, Pablo Gálvez-Ruiz, Moisés Grimaldi-Puyana, Salvador Angosto, Jesús Fernández-Gavira, M. Rocío Bohórquez

**Affiliations:** 1 Department of Physical Education and Sports, Universidad de Sevilla, 41013 Seville, Spain; jeronimo@us.es (J.G.-F.); mgrimaldi@us.es (M.G.-P.); jesusfgavira@us.es (J.F.-G.); 2 Department of Physical Education and Sports, Universidad Internacional de Valencia, 46002 Valencia, Spain; 3 Department of Physical Education and Sports, University of Murcia, 30071 Murcia, Spain; salvador.a.s@um.es; 4 Department of Social Psychology, University of Sevilla, 41013 Seville, Spain; rociobohorquez@us.es

**Keywords:** e-lifestyles, physical activity, intention to use, fitness services, fitness app

## Abstract

E-Lifestyles are individual forms of behavior in the digital environment that reflect the values, activities, interests, and opinions of consumers. Likewise, fitness Apps are considered technological tools for promoting physical activity online. Although there are studies related to sports lifestyles, it has not been analyzed yet how e-lifestyles are related to the use of fitness Apps. Based on this, this study represents a step to clarify how e-lifestyles influence different relationships with perceived ease of use, perceived usefulness, attitude, and intentions to use Fitness Apps. Therefore, the objective of the study was to analyze the relationship between the e-lifestyles of consumers of Boutique fitness centers and their relationship with the perceived ease of use, the perceived usefulness, the attitude, and the intention to use Fitness Apps. The sample was 591 customers (378 women and 213 men) of 25 Boutique fitness centers. An online questionnaire was used for data collection. Data was analyzed with confirmatory factor analysis and structural equation model. Findings provide an insight into the importance of e-lifestyles in the intention of using fitness Apps and therefore in promoting physical activity through online fitness services. The results showed positive relationships between e-lifestyles, perceived ease of use, perceived usefulness and attitude toward fitness Apps. Finally, the attitude toward fitness Apps offered a very high predictive value on use intention. This study provides a better understanding of consumer´s intention to use fitness Apps. The conclusions and recommendations for sports managers of fitness centers highlight the importance of e-lifestyles as a predecessor for the use of fitness Apps.

## 1. Introduction

The sports sector has become a highly competitive and sometimes saturated market in some of its areas, like physical education teachers or public administration [1]. This influences the development of strategies that allow organizations to generate a differential advantage over their competitors, positioning them in a specific offer within the market [2]. Specifically, the fitness sector, framed in the sports sector, is constantly being updated, and new models of specialized gyms are emerging. These new fitness facilities are adapting to new business proposals that tend toward professionalism, cost control, and the ability to increase income from various sources, called Boutique fitness centers [3,4].

Boutique fitness centers are characterized by being small facilities (between 100 and 500 m^2^) with lower economic investments than other models of fitness centers and whose objective is to offer very specific services and activities aimed at a specific public and population sector [5,6]. The main activity of Boutique fitness centers is the fitness activity taught by a personal trainer, aimed at a small group—no more than ten people—using cardiovascular equipment, strength, and accessories, with an average of between 200 to 400 customers and with a monthly fee of between 50–120 euros [6]. These fitness facilities are currently one of the main fitness trends in Spain, having increased from four centers in 2010 to 305 in 2017 [6,7], and one of the top ten references worldwide [8].

Looking for new opportunities and business models in the fitness sector in general and Boutique fitness centers in particular, technology-based service delivery is growing exponentially [6,7]. The reasons behind this increase are the consumer’s desire for self-service, the reduction of costs due to automation, and greater organizational efficiency [9,10]. In this sense, the determination of the factors that may influence the adoption of the use of new technologies such as the fitness Apps associated with Boutique fitness centers is essential for their design, development and implementation. Likewise, its importance lies in the fact that the fitness Apps favor a greater participation in physical activities and, therefore, a more active and healthy population [11].

One of the factors that has been determining for the use intention of mobile apps has been e-lifestyles [12]. However, this relationship between e-lifestyles and intention to use Apps in fitness contexts has not yet been sufficiently investigated. For this reason, the objective of this work is to analyze the relationship between e-lifestyles, the perceived ease of use of fitness Apps, the perceived usefulness of fitness Apps, the attitude, and the intentions of use of fitness Apps in Boutique fitness centers.

## 2. Literature Review

### 2.1. Consumer Lifestyles and E-Lifestyles

Lifestyles are distinctive patterns of behavior; understanding these patterns in a broad and global sense [13] involves people’s physical and psychological environments [14]. Thus, they are based on a large number of individual demographic, domestic, leisure, and professional variables [15].

These behavior patterns are specified in the way in which people invest their time and money, in their interests, and in their vital priorities, among other aspects [16,17] having an impact on consumer health [18]. Specifically, regarding consumer behavior, the decisions made are determined by cultural patterns [19], purchasing power, personality, motivation, or family history [20]. The segmentation of lifestyles is considered an extension of psychographic segmentation based on the study of consumer interests, opinions, and daily activities [21].

According to the literature, some authors used sociodemographic variables such as age, gender, education, or income level to classify mobile device users [22,23]. However, González and Bello [24] and Yang [10] state that the sociodemographic variables are limited for the study of users’ consumption patterns. Thus, the segmentation of consumers according to their lifestyles helps to know and predict their consumption behaviors [25,26]. Zaheer and Kline [14] highlight the importance of lifestyle segmentation due to its limited literature [14,20,21,27,28]. For this reason, it is possible to find works that have used the segmentation of the population according to their lifestyles in sectors as diverse as tourism, organic products, shopping centers, cinema, the financial sector [29], world fashion [30], the pharmaceutical industry [31], and even the sports sector [32]. Specifically, Suresh et al. [32] claim to be able to determine the loyalty of external clients of wellness centers through marketing strategies based on segmentation according to lifestyles.

In the context of technology consumption, Hoon et al. [33] postulated that electronic lifestyles could differ from traditional lifestyles and therefore should be studied specifically and independently of the former. Technological innovations and the evolution of smartphones have generated a new dimension of electronic lifestyle associated with products and services based on information and communication technologies [34]. For Hoon et al. [33] and Yu [2], e-lifestyles are individual forms of behavior in the digital environment that reflect the values, activities, interests, and opinions of consumers. In line with this recommendation, Lee et al. [27] identified four e-lifestyle factors: fashion consciousness, leisure orientation, Internet involvement, and e-shopping preferences. Fashion consciousness is defined as the level of involvement of a person with fashion and prevailing trends [35]. This implication has been shown to affect decision making regarding the consumption of technological products [36,37]. Leisure orientation reflects patterns of the active pursuit of intrinsically motivating activities to spend free time on. For its part, Internet involvement reflects behavior patterns focused on what is digital, so that the person makes extensive use of the internet both for their work (for example, searching for information or sending a large number of emails) and for the rest of personal facets (for example, using online entertainment systems) [27,34]. Finally, e-shopping preference is related to the tendency of certain consumers to purchase products and services digitally rather than through traditional systems [38].

Although these e-lifestyles are changing consumption patterns, it is necessary to understand what theories are related to understanding the associated variables. In fact, these relationships between e-lifestyles and digital consumption have been strongly linked to different technological theories, helping to understand people’s behavior toward information and communication technologies and their use.

### 2.2. Technology Acceptance Model

The Theory of Reasoned Action (TRA) [39] explains the relationship between people’s attitudes, their behavioral intention, and their actual behavior, the latter influenced by the subjective social norm. Based on this theory, Davis [40] developed the Technology Acceptance Model (TAM) with the aim of explaining how consumers use and accept new information technologies. The TAM relates two concrete base factors, the perceived ease of use (PEU), and the perceived usefulness (PU), with the consequent intention of behavior and the subsequent real behavior of the individual. PEU can be defined as the cognitive and deliberative level that is required of the individual to learn to use technology [41]. That is, the effort that the user must make—or not—to use the technology [42]. PU is defined as the degree to which people believe that a certain system will help perform a certain task [43], usually the degree to which it will simplify their work [42]. The importance of this model is the positive relationship between its variables and its influence on attitudes toward technology and the intention to use it [44,45].

For this reason, the TAM has been one of the most traditionally used models in professional settings thanks to its great robustness and applicability [9,46], paying special attention to the utilitarian aspect of the technology [47] and with the intention of understanding the consumer’s intention to use it [48]. The model has shown its utility by explaining the use of mobile technology in different contexts such as finance, instant messaging, healthcare, gambling, and tourism [9].

In the sports sector, there are some studies that have analyzed these variables, finding relationships between PEU and PU [49,50], PEU and PU on the intention of use [25,49,51], or the intention of use and the actual use of the App [12]. Likewise, they have been analyzed in different sport contexts, such as sports websites [52], fitness Apps [49], sports products [53], sport teams Apps [51], or smartphone use for sports consumption [50].

Finally, the model has been influenced by other external variables, such as social norms [49], health awareness [54], commitment to or participation in sport [50], and entertainment [55], and sociodemographic variables, such as gender, age, or frequency of use [53]. In this way, research with TAM has determined that PEU and PU are influenced by different external variables. However, it is not known whether lifestyles or e-lifestyles could also affect these relationships in the fitness context.

### 2.3. Lifestyles, E-lifestyles, and TAM Relationships

Coursaris and Van Osch [15] state that the relationship between e-lifestyles and the intention to use technologies has not been widely studied. Still, there are some works that have linked e-lifestyles with the use and acceptance of new technologies, using TAM as a theoretical basis (i.e., [27,42,56]). Thus, it has been suggested that e-lifestyles determine attitudes that can affect the intention to use information and communication technologies [15,27,37,57].

Kim and Lee [42] relate e-lifestyles with motivation and the intention to use in Apps advertising, finding four different profiles of consumers according to their e-lifestyle, where motivation significantly influenced PEU and PU as well as the future intention of use. For their part, Pan et al. [56] indicated that lifestyles can also influence consumer adoption intention and behavior through factors such as aesthetics, compatibility, or work.

Specifically, e-lifestyle characterized as fashion consciousness has been shown to have a positive impact on the PEU and PU of consumers of technological products [27,56]. Furthermore, it has been shown to have a positive impact on the intention to purchase high-end technology products [27] as well as digital services [37]. Regarding the leisure orientation lifestyle, consumers with this e-lifestyle perceived technological products as more useful for creating intrinsically motivating moments than consumers with low leisure orientation, correlating with the intention of adopting high-end technological products [27].

Thus, an e-lifestyle highly involved with the use of the Internet positively correlates with the PEU and the PU of online banking consumers [58]. Along these lines, consumers with a high technological knowledge better understand the product and are more likely to be involved in the purchase of these devices [59]. Finally, e-lifestyles and the trend toward online shopping have changed consumer behaviors [60]. Hence, Lee et al. [27] point out that an e-lifestyle tending to online shopping positively correlates with the PU of technological devices.

**Figure 1:**
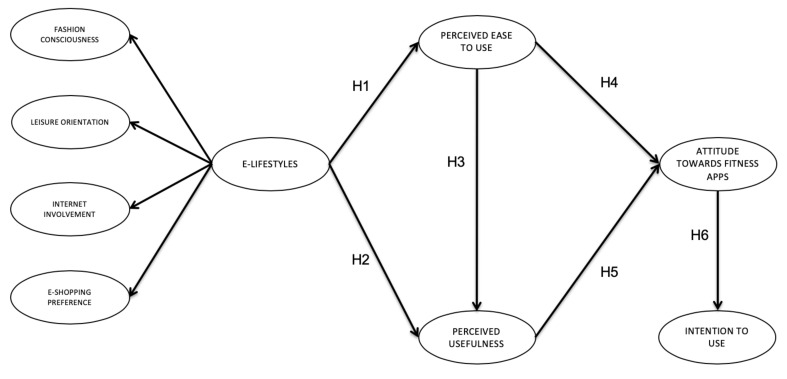
Proposed model.

Based on the literature review, we developed the following five hypotheses (Figure 1):

**Hypothesis** **1.** *There is a direct and positive relationship between e-lifestyle and PEU in Boutique fitness centers customers.*

**Hypothesis** **2.** *There is a direct and positive relationship between e-lifestyle and PU in Boutique fitness centers customers.*

**Hypothesis** **3.** *There is a direct and positive relationship between PEU and PU in Boutique fitness centers customers.*

**Hypothesis** **4.** *There is a direct and positive relationship between PEU and attitude toward fitness Apps in Boutique fitness centers customers.*

**Hypothesis** **5.** *There is a direct and positive relationship between PU and attitude toward fitness Apps in Boutique fitness centers customers.*

**Hypothesis** **6.** *There is a direct and positive relationship between attitude toward fitness Apps and intention to use in Boutique fitness centers customers.*

## 3. Materials and Methods

### 3.1. Study Context

According to different studies and international organizations, the fitness sector is a growing market [8]. Among the different fitness business models that exist in the industry, in recent years low-cost fitness centers have been those that have had the greatest increase in the volume of fitness facilities and consumers. However, Europe Active [61] has reported that there has been a notable increase in Boutique fitness center chains that have created a new situation in the fitness sector. These chains of Boutique fitness centers are characterized by the personalization of the service, they are social, they are trendy, and they help members achieve fast results [3].

Spain is one of the countries with the highest market penetration in the fitness sector [8], having created chains of Boutique fitness centers. Among them, Sano Center was created in 2014 and currently has more than 50 centers located in Spain and Mexico. Its positioning is due to the high personalization of its services, a highly individualized study of the client’s objectives, and a team of professionals specialized in physical activity, who design programs based on the physical characteristics of the client and help users find their objectives in a personalized way. To track and prescribe training in a digital format, the chain of Boutique fitness centers uses an App from a university spin-off called Fitbe. This App allows the visualization of the exercises, the realization of directed classes in streaming and the interaction with clients through video calls.

### 3.2. Participants

The sample is made up of 591 customers (378 women and 213 men) from the Boutique fitness center chain, Sano Center. The inclusion criteria to participate in the study was that the customers had downloaded the specific fitness App from Sano Center. Of the sample, 4.6% (*n* = 27) were under 20 years old, 25.7% (*n* = 152) between 21 and 30 years old, 34.2% (*n* = 202) between 31 and 40 years old, 28.0% (*n* = 168) between 41 and 50 years old, and 7.1% (*n* = 42) were over 50 years old. With respect to length of membership, 26.4% (*n* = 156) had been less than three months as a customer, 18.8% (*n* = 111) between three and six months, 27.9% (*n* = 165) between six and twelve months, and 26.9% (*n* = 159) over one year. In turn, it is worth noting that 56% (*n* = 331) attend twice a week and 36.7% (*n* = 217) three times a week.

### 3.3. Measures

A questionnaire instrument was developed using prior research [9,38,51,62]. The questionnaire included demographic questions and a total of 34 items grouped into two sections. The first section consisted of scales to measure customer e-lifestyles [27]. Three items measured fashion consciousness (e.g., “design is the most important factor in choosing electronic products”), four measured leisure orientation (e.g., “I thoroughly enjoy my leisure time”), Internet involvement was measured by four items (e.g., “I spend less time watching TV because of the Internet”), and seven items measured e-shopping preference (e.g., “I enjoy buying things on the Internet”). The customers had to evaluate the perception of the specific fitness App from Sano Center. Thus, the second section consisted of 12 items proposed by Kim and Chiu [63] to evaluate perceived ease of use (4 items; e.g., “fitness Apps are easy to use”), perceived usefulness (4 items; e.g., “using fitness Apps improves my exercise experience”), and intention to use (4 items; e.g., “I will use fitness Apps on a regular basis in the future”). To evaluate attitude toward Fitness App, we used the scale proposed by Rivera et al. [9] with 4 items (e.g., “using fitness Apps is a good idea”). A five-point Likert scale with a range from “completely disagree” (1) to “completely agree” (5) was used for possible responses.

### 3.4. Procedure

The Boutique fitness center chain, Sano Center, was contacted to inform them of the study and the intended objectives. After two meetings with the general management and the sports and marketing managers, it was decided to ask the managers of each fitness facility if they wanted to participate in the study. After the approval of 25 centers belonging to the Boutique fitness centers chain, the online questionnaire was sent to each of the facilities. Finally, each director sent the questionnaire to his/her customers. Google Forms was used to collect information. All the participants were guaranteed personal confidentiality and informed of the importance of their honesty in answering the questions and the voluntary nature of participating in the research. Once all the information was collected, it was combined into a single database to be able to use it for data analysis. The information collection period lasted three months.

### 3.5. Data Analysis

We calculated descriptive statistics (means and standard deviation). The normality of the data (univariate skewness and kurtosis), with values smaller than the criterion 3 and 7, respectively [64], supported the normality for structural equation model (SEM) analysis. We performed a maximum likelihood method of estimation for structural equation modeling using the AMOS 21.0 (21.0, IBM SPSS, Chicago, IL, USA), procedure recommended by Joreskog and Sorbom [65] for conducting path analysis. First, we conducted confirmatory factor analysis (CFA) to test the psychometric properties for the measurement model. Second, we conducted a SEM that analyzed the predicted hypothesized relationships between the variables for the present study. In the above SEM (Figure 1), four reflective variables and corresponding observing variables constitute the intention of using the evaluation model in the context of the present study. For both analyses, we used the following goodness-of-fit indexes: the ratio of the chi-square to its degrees of freedom (*χ*^2^/df), the Root Mean Square Error of Approximation (RMSEA) and the respective confidence interval (90% CI), the Comparative Fit Index (CFI), the Tucker–Lewis Index (TLI), and the Parsimony Comparative Fit Index (PCFI). For these indexes, the following cut-off values were adopted: *χ*^2^/df ≤ 3 [66], RMSEA ≤ 0.08 [67,68], CFI and TLI ≥ 0.90 [69,70], and PCFI ≥ 0.80 [71].

Internal consistency was calculated via composite reliability, adopting 0.70 as the cut-off value [69,72]. Convergent validity was examined through an average variance extracted calculation (AVE ≥ 0.50; [72,73]), while discriminant validity was established when the AVE for each construct exceeded the squared correlations between that construct and any other [72,74].

## 4. Results

### 4.1. Descriptive Analysis

There were no missing values in the data and an item-level descriptive statistics showed no deviations from univariate normality in any of the items (all the skewness and kurtosis values were lower than 3 and 7, respectively). The participants’ assessments exceeded the midpoint of the scale with the exception of items Leisure Orientation 4 (*M* = 1.64), Internet Involvement 10 (*M* = 1.69), E-Shopping Preference 17 (*M* = 2.03), all belonging to the e-lifestyle scale. 

### 4.2. Measurement Model Assessment

Confirmatory factor analysis was performed on the measurement model and found to have excellent goodness-of-fit statistics: *χ*^2^(499) = 1083.90 (*p* = 0.000); *χ*^2^/df = 2.17; RMSEA = 0.060 (CI = 0.055, 0.065); CFI = 0.93; TLI = 0.92; PCFI = 0.82. The χ^2^/df value was situated below the minimum acceptable value of 3.0. The RMSEA index offered a good adjustment obtaining an index of 0.08. The CFI and TLI values were greater than the minimum recommended threshold of 0.90, and the PCFI index was above the good adjustment threshold 0.80. This is satisfactory evidence of proportional adjustment.

The size of the factor loading is a criterion used to evaluate the reliability of the indicator with the constructs it intends to measure [75]. For this reason, the items were maintained with a factorial loading (λ) greater than the conservative threshold of 0.50 [76], and items not loading properly were eliminated. All the values, except three items (Leisure Orientation 4: λ = 0.12; Internet Involvement 10: λ = 0.24; E-Shopping Preference 17: λ = 0.34), were greater than 0.50, so when eliminating them, the overall fit indices indicated the robustness of the resulting measurement model: *χ*^2^(406) = 878.79 (*p* = 0.000); *χ*^2^/df = 2.16; RMSEA = 0.059 (CI = 0.054, 0.065); CFI = 0.94; TLI = 0.93; PCFI = 0.83. All the factor loadings were statistically significant (*p* < 0.01), and also the z-values ranged from 10.80 to 34.47, indicating that the items accurately captured their respective factors [77]. In this sense, evidence of the measures’ validity is provided by the fact that all factor loadings are significant and above 0.5, suggesting high levels of internal consistency and adequate item reliability [76] as seen in Table 1.

The composite reliability (CR) for all the constructs ranged from 0.75 to 0.97 (values above the recommended 0.70), and average variance extracted (AVE) ranged from 0.50 to 0.89 (greater than the prescribed 0.50). As shown in Table 2, fashion consciousness has lower mean scores, while leisure orientation presents the highest average of the four e-lifestyle factors. The discriminant validity of the measures was accepted given that the square correlations between each construct and any others were lower than de AVE values for each construct [72].

### 4.3. Structural Model Assessment

We estimated the hypothesized model and the standardized regression weights for the causal paths as presented in Table 3. The results indicate support for all the causal relationships except H2 and H4, along with excellent goodness-of-fit for the causal model: *χ*^2^(424) = 942.59 (*p* = 0.000); *χ*^2^/df = 2.23; RMSEA = 0.061 (CI = 0.056, 0.066); CFI = 0.93; TLI = 0.93; PCFI = 0.85. The hypothetical model established that e-lifestyles were positive and significant predictors of the perceived ease of use but were not significant concerning the perceived usefulness. However, the perceived usefulness was shown to be a strong predictor on the attitude toward mobile apps, which does not occur with perceived ease of use. Finally, the attitude toward mobile apps offered a very high predictive value on intention to use.

### 4.4. Measurement Invariance

Regarding the invariance of the measurement model across genders, the results showed that it is invariant. Furthermore, as demonstrated in Table 4, ΔCFI, ΔRMSEA, and ΔNNFI were acceptable according to [78] recommendations for measurement invariance. Despite measurement invariance in our results, it is possible to observe that the residual invariance score calculated by ΔCFI in all the models was >0.01 [78,79].

## 5. Discussion

The sports industry, and in particular the fitness sector, has always been linked to the promotion of healthy habits and the promotion of physical activity. Specifically, the fitness sector aims to provide fitness services that foster people’s quality of life. These fitness services have been offered from business models that back low-cost [80] to highly personalized business models that are conceptualized as Boutique fitness centers [3,4]. Likewise, until now, fitness services have been eminently face-to-face, but the new situation caused by the Covid-19 pandemic has meant that fitness services can also be online, and therefore, technology can become a great ally for the promotion of physical activity. In fact, people’s consumption has been transformed by a more technological lifestyle that could therefore change the perspectives and the promotion of physical activity [81]. That is why e-lifestyles and the promotion of physical activity could be closely linked. Thus, the promotion of physical activity is being offered from online fitness services through mobile Apps [82]. With this premise, the objective of this work was to analyze the relationship between the e-lifestyles of consumers of Boutique fitness centers and the perceived ease of use, the perceived usefulness, the attitude, and the intention to use fitness Apps.

Some authors have indicated that lifestyles can be summarized in behavioral patterns linked to the investment of time, money, or interests, which could in turn have an impact on consumer health [17,18]. Specifically, e-lifestyles refer to people’s behaviors in which interests, activities and opinions are observed in the digital environment [28,33]. Thus, the findings of this study have helped to understand how e-lifestyles are linked to technological behaviors in relation to fitness Apps and could therefore favor the prediction of fitness consumption behaviors [26]. Similarly, these results have highlighted the importance of the analysis of e-lifestyles, which are currently changing fast and are therefore necessary to analyze [83]. Thus, among the theories with the greatest recognition in relation to the understanding of the intention to use technologies, this study opted for the TAM [40]. The importance of the model lies in the acceptance by the literature of the positive relationship between ease of use and the perceived usefulness regarding attitudes toward technology and the intention to use them. In fact, it is a model that presents great robustness and applicability in numerous sectors [9].

In particular, the findings found in this study have shown a positive relationship between e-lifestyles and the perceived ease of use fitness Apps. Hence, these results have coincided with works carried out in other sectors [27,56,58], which makes this study the first to corroborate such findings in the sports sector and, more specifically, in the fitness sector. In fact, Lee et al. [27] stated that there was a positive relationship between e-lifestyles and the intention to use Apps in high-technology products, as Pan et al. [56] and Boateng et al. [58] showed for online banking. For this reason, this study reduces the research gap in the sports management literature, as it stated that e-lifestyles have a positive effect on the intentions to use fitness Apps. That is why this research, firstly, gives an important role to e-lifestyles as predecessors of the mobile applications´ use, and secondly, it provides knowledge of this relationship in the fitness sector, in particular in relation to the use intention of fitness Apps. Thus, so far, there are no studies that have shown these relationships in the fitness sector, even though it is considered as a sector in global growth [8].

However, the results did not coincide with previous studies in relation to their influence on the perceived usefulness [27,58], which suggests that the usefulness of fitness Apps is not conditioned by e-lifestyles. In fact, these findings show that e-lifestyles linked to online shopping preferences, the use of technology as an extensive aspect of daily life or work, or the level of involvement by a technological trend, would directly influence the perceived ease of use Fitness Apps but not the final perceived usefulness offered by these virtual tools.

Likewise, this study has shown that if e-lifestyles are directly related to the perceived ease of use fitness Apps, they would later also be related to the perceived usefulness of the Apps. These relationships therefore confirm previous studies in the sports sector in which perceived ease of use has a positive and direct relationship with the perceived usefulness of fitness Apps [49]. That is why the perceived ease of use would mediate between e-lifestyles and the perceived usefulness of fitness Apps. In fact, the findings found after the analyses are interesting, since no positive relationships were found between perceived ease of use and attitudes toward fitness Apps. For this reason, the results do not corroborate what was previously studied [49,51], so it is suggested that e-lifestyles could have an indirect impact on these relationships. Thus, previous studies that affirmed positive relationships between perceived usefulness and attitudes toward fitness Apps were corroborated by this work, also resulting in a positive relationship with the intentions of use [49,51].

These results have contributed to a gap that existed in the academic literature on the history of the use of fitness Apps. In fact, authors such as Ha et al. [50] showed that there were no research works that had investigated the factors or behaviors that conducted sports consumers to use sports Apps. Thus, as with previous authors [27,37,57], this study’s findings have shown that the use intentions of fitness Apps could be conditioned initially by e-lifestyles and that perceived ease of use and the perceived usefulness would mediate the attitudes and intentions of using Fitness Apps.

### 5.1. Practical Implications

As practical applications, this study confirms the prominent role of e-lifestyles as predictors of fitness app adoption. Therefore, it is the first related study that highlights the importance of values and behaviors in the digital environment for people to use fitness Apps. Understanding the effects of e-lifestyles on the adoption of fitness Apps could allow fitness centers to emphasize the benefits’ characteristics of practicing online fitness services. In fact, this aspect is very important due to the long periods of confinement that have occurred due to the Covid-19 pandemic. The results of the study have provided knowledge that the use of fitness Apps, and therefore the consequences of practicing physical activity through these technological tools, is influenced by technological preferences and consumers’ knowledge of them. For this reason, if the fitness centers’ managers use Fitness Apps in order to prescribe and control the fitness services, they should train and adapt consumers to technological habits. For example, advertising campaigns for fitness centers could highlight the importance and benefits of using technology in fitness centers. This fact would provoke changes in e-lifestyles, favoring their involvement in the ease of use of fitness Apps. Thus, it is logical to think that consumers with more developed e-lifestyles also have a greater perception of ease of use and therefore lead to a greater intention to use fitness Apps. Therefore, the promotion of online fitness services ought to be associated with strategies that increase perceived ease of use and perceived usefulness, since this would lead to a greater use of these sports technologies and therefore a greater promotion of online fitness services.

### 5.2. Limitations and Future Lines of Research

Like all research work, this study has a number of limitations. Firstly, the sample used belongs to a business model in the fitness sector where the ages included were under 50 years old. This situation could result in different behaviors and e-lifestyles to other older population groups, and therefore, the relationships between the variables could also be different. The results have also been tested from a participants´ sample from a specific country and business model (Boutique). This fact could interfere with the generalization of the findings due not only to the digital culture of the participants themselves but also to the use of the fitness app in the fitness chain where the study hypotheses have been studied. Similarly, the model tested has used e-lifestyles as a second order variable, which could reduce the resulting information. That is why although it is one of the first investigations to study these relationships in fitness Apps, the results could be basic in terms of the relationships between the variables analyzed. Likewise, the relationships between e-lifestyles and the variables proposed in the TAM model have been tested. Although the TAM model has been the most referenced and used in the fitness sector [84], it could be improved by the inclusion of other variables. These limitations help to understand what the future lines of research could be. In particular, the model tested could be applied to other business models in the fitness sector and therefore characterized by different population groups. Possibly, the e-lifestyles of the youngest would be different from those of the oldest, and therefore, the relationships obtained could differ. Likewise, it would be interesting to investigate separately the relationships of the different e-lifestyles (fashion consciousness, leisure orientation, internet involvement, and e-shopping preference) and perceived ease of use and perceived usefulness, since it has been seen that such relationships could be modified separately. Furthermore, the separate analysis of e-lifestyles could help to understand consumer segmentations and their intention to use Fitness Apps. Finally, an analysis with other models that predict the use of technologies in the fitness industry could help to comprehend the relationship of e-lifestyles with other variables that predict the intention of using fitness Apps and would therefore promote physical activity.

## 6. Conclusions

This study analyzed the relationships between e-lifestyles and the final intention to use fitness Apps. To do so, the model tested included mediating variables such as perceived ease of use, perceived usefulness, and attitudes toward fitness Apps. Having carried out analyses, and in light of the findings, it can be concluded that e-lifestyles influence perceived ease of use, and this in turn influences perceived usefulness, which likewise influences attitudes toward and intention to use. These conclusions provide knowledge about the importance of e-lifestyles in the intentions of using fitness Apps and therefore in promoting physical activity through online fitness services.

## Figures and Tables

**Table 1:** Normality (univariate skewness and kurtosis), factor loadings in confirmatory factor analysis (CFA), composite reliability (CR), and average variance extracted (AVE).

	Normality US/K	CFA Loadings	CR	AVE
**Factors/Items**				
**Fashion Consciousness**			0.81	0.59
FA1. Design is the most important factor in choosing electronic products	−0.18/−0.93	0.68		
FA2. When I must choose between the two I usually buy an electronic device	−0.31/−0.82	0.93		
FA3. I often buy the latest model in electronic products	−0.05/−1.13	0.66		
**Leisure Orientation**			0.75	0.50
LO5. I would rather enjoy leisure time than work hard	−0.35/−0.99	0.61		
LO6. Leisure is worth the extra money spent for it	−1.19/0.89	0.75		
LO7. I thoroughly enjoy my leisure time	−1.43/1.94	0.75		
**Internet Involvement**			0.75	0.51
II8. I spend less time watching TV because of the Internet	−0.48/−0.84	0.68		
II9. I am doing more shopping on the Internet than before	−0.79/−0.61	0.76		
II11. I trust information on Web sites that I have heard about	−0.04/−0.56	0.69		
**E-Shopping Preference**			0.80	0.50
ESP12. I think online buying is a novel, fun way to shop	−0.45/−0.45	0.78		
ESP13. E-shopping is easier than local shopping	−0.06/−0.96	0.69		
ESP14. I like browsing on the Internet	−1.02/0.53	0.71		
ESP15. I think e-shopping offers lower prices than local stores	−0.49/−0.42	0.63		
ESP16. I enjoy buying things on the Internet	−0.23/−0.79	0.77		
ESP18. I think e-shopping offers a better selection than local stores	−0.11/−0.89	0.60		
**Perceived Ease of Use**			0.97	0.89
PEU1. Fitness Apps are easy to use	−0.84/0.58	0.89		
PEU2. Learning to use fitness Apps is easy	−1.03/1.20	0.92		
PEU3. Interaction with fitness Apps is clear and understandable	−0.85/0.63	0.96		
PEU4. It is easy to interact with fitness Apps	−0.84/0.71	0.97		
**Perceived Usefulness**			0.93	0.77
PU1. Using fitness Apps improves my exercise experience	−0.32/−0.33	0.83		
PU2. Using fitness Apps enhances my effectiveness in doing exercises	−0.08/−0.37	0.86		
PU3. Using fitness Apps increases my productivity in doing exercises	−0.09/−0.45	0.93		
PU4. Using fitness Apps is useful for doing exercises	−0.32/−0.46	0.87		
**Attitude Toward Fitness Apps**			0.91	0.71
AT1. Using fitness Apps is a good idea	−0.80/0.71	0.78		
AT2. I intend to use fitness Apps in my fitness center	−0.51/−0.39	0.82		
AT3. Fitness Apps make the physical activity more interesting	−0.25/−0.61	0.87		
AT4. I like doing physical activity with fitness Apps	−0.13/−0.72	0.87		
**Intention to Use**			0.96	0.86
IU1. I will use fitness Apps on a regular basis in the future	−0.19/−0.58	0.94		
IU2. I will frequently use fitness Apps in the future	−0.09/−0.49	0.94		
IU3. Assuming I have access to fitness Apps, I intend to use them	−0.55/−0.23	0.92		
IU4. Given that I have access to fitness Apps, I predict that I would use them	−0.48/−0.24	0.91		

Note. FA = Fashion Consciousness; LO = Leisure Orientation; II = Internet Involvement; ESP = E-Shopping Preference; PEU = Perceived Ease of Use; PU = Perceived Usefulness; AT = Attitude Toward Fitness Apps.

**Table 2:** Mean, standard deviation, convergent and discriminant validity.

Factors	M	SD	AVE	FA	LO	II	ESP	PEU	PU	AT	IU
FA	3.02	0.97	0.59	1.0							
LO	3.79	0.87	0.50	0.09	1.0						
II	3.30	0.90	0.51	0.09	0.49	1.0					
ESP	3.33	0.86	0.50	0.15	0.29	0.47	1.0				
PEU	3.75	0.92	0.89	0.06	0.11	0.08	0.12	1.0			
PU	3.16	0.95	0.77	0.08	0.07	0.06	0.10	0.26	1.0		
AT	3.36	0.95	0.71	0.09	0.10	0.07	0.14	0.21	0.68	1.0	
IU	3.29	1.02	0.86	0.06	0.11	0.11	0.13	0.18	0.44	0.64	1.0

Note. M = mean; SD = standard deviation; AVE = average variance extracted; FA = fashion consciousness; LO = leisure orientation; II = internet involvement; ESP = E-shopping preference; PEU = perceived ease of use; PU = perceived usefulness; AT = attitude toward fitness apps; IU = intention to use.

**Table 3:** Standardized regression weights for the causal paths.

Predictor Variables	Criterion Variables	Hypothesized Relationship	Standardized Coefficient (β)	Results
e-Lifestyles	Perceived ease of use	H1	0.373 ***	Supported
e-Lifestyles	Perceived usefulness	H2	0.178	Not supported
Perceived ease of use	Perceived usefulness	H3	0.452 ***	Supported
Perceived ease of use	Attitude toward mobile apps	H4	0.061	Not supported
Perceived usefulness	Attitude toward mobile apps	H5	0.783 ***	Supported
Attitude toward mobile apps	Intention to use	H6	0.872 ***	Supported

Note. *** *p*-value < 0.001.

**Table 4:** Goodness-of-fit indexes of measurement invariance across genders.

Models	χ^2^ (df)	Δχ^2^ (df)	p	CFI	ΔCFI	SRMR	ΔSRMR	RMSEA	ΔRMSEA
Measurement invariance across genders									
CI	1612.95 (848)			0.910		0.077		0.052	
MI	1653.38 (871)	40.43 (23)	<0.001	0.908	0.002	0.072	0.005	0.052	0.000
SI	1662.50 (880)	49.55 (32)	<0.001	0.908	0.002	0.071	0.006	0.052	0.000
RI	1676.35 (889)	63.40 (41)	<0.001	0.907	0.003	0.068	0.009	0.052	0.000

Note. χ^2^ = chi-squared: df = degrees of freedom; Δχ^2^ = differences in the value of chi-squared; CFI = Comparative Fit Index; SRMR = Standardized Root Mean Square Residual; RMSEA = Root Mean Square Error of Approximation; CI = configural invariance; MI = measurement invariance; SI = structural invariance; RI = residual invariance.
